# Enhancing Presurgical Infant Orthopedic Appliances: Characterization, Mechanics, and Biofilm Inhibition of a Novel Chlorhexidine-Halloysite Nanotube-Modified PMMA

**DOI:** 10.1155/2024/6281972

**Published:** 2024-05-10

**Authors:** Nadia Al Ansari, Mushriq Abid

**Affiliations:** ^1^Department of Orthodontics, Al Rafidain University College, Baghdad, Iraq; ^2^Department of Orthodontic, College of Dentistry, University of Baghdad, Baghdad, Iraq

## Abstract

**Objectives:**

This *in vitro* study aimed to develop a novel nanocomposite acrylic resin with inherent antimicrobial properties. This study evaluated its effectiveness against microbial biofilm formation, while also assessing its physical and mechanical properties.

**Methods:**

Polymethylmethacrylate (PMMA) was modified with four different concentrations of chlorhexidine halloysite nanotubes (CHX-HNTs): 1%, 1.5%, 3%, and 4.5 wt.% by weight, along with a control group (0 wt.% CHX-HNTs). The biofilm inhibition ability of the modified CHX-HNTs acrylic against *Candida albicans*, *Staphylococcus aureus*, *Streptococcus pneumoniae*, and *Streptococcus agalactiae* was assessed using microtiter biofilm test. In addition, ten samples from each group were then tested for flexural strength, surface roughness, and hardness. Statistical analysis was performed using one-way ANOVA and Tukey's test for comparison (*P* < 0.05).

**Results:**

CHX-HNTs effectively reduced the adhesion of *Candida albicans* and bacteria to the PMMA in a dose-dependent manner. The higher the concentration of CHX-HNTs, the greater the reduction in microbial adhesion, with the highest concentration (4.5 wt.%) showing the most significant effect with inhibition rates ≥98%. The addition of CHX-HNTs at any tested concentration (1%, 1.5%, 3%, and 4.5 wt.%) did not cause any statistically significant difference in the flexural strength, surface roughness, or hardness of the PMMA compared to the control group.

**Conclusions:**

The novel integration of CHX-HNT fillers shows promising results as an effective biofilm inhibitor on acrylic appliances. This new approach has the potential to successfully control infectious diseases without negatively affecting the mechanical properties of the acrylic resin. *Clinical Relevance*. The integration of CHX-HNTs into presurgical infant orthopedic appliances should be thoroughly assessed as a promising preventive measure to mitigate microbial infections. This evaluation holds significant potential for controlling infectious diseases among infants with cleft lip and palate, thereby offering a valuable contribution to their overall well-being.

## 1. Introduction

Cleft lip and palate (CLP) present a significant surgical challenge due to facial asymmetry, tissue absence, and the size of the gap. Since the 1950s, orthodontists and surgeons have collaborated on developing presurgical infant orthopedic (PSIO) to align the soft tissue and osseous structures before surgery [1, 2]. PSIO procedures, particularly nasoalveolar molding (NAM), have become widely adopted worldwide over the past 40 years [3–6]. In the United States, 71% of centers use some form of PSIO device before cleft lip surgery, with NAM being the most common approach (55%) [6].

While polymethyl methacrylate (PMMA) is widely used in dentures and orthodontic appliances due to its affordability and aesthetics, its porous surface and the fluctuating oral environment promote bacterial and fungal growth, leading to plaque buildup [7–12]. These concerns are even more significant for serious pathogens such as methicillin-resistant *Staphylococcus aureus*, which have been found on such appliances and can potentially lead to a local or systemic infection [13, 14]. Oral microorganisms derived from removable acrylic appliances have been implicated in bacterial endocarditis, pneumonia, chronic obstructive pulmonary disease, and gastrointestinal infections [15]. Maintaining proper hygiene with these removable appliances is especially difficult for children and disabled patients [16]. Therefore, integrating antimicrobial properties directly into PSIO appliances presents a promising solution to minimize plaque formation and improve overall oral hygiene.

Nanotechnology has emerged as a powerful tool for developing materials with superior biological and mechanical properties [17, 18]. Halloysite nanotubes (HNTs), a natural nanomaterial similar to kaolin and derived from weathered alumina silicate clays, have gained particular interest for their drug delivery capabilities [19]. Studies have demonstrated successful encapsulation of various compounds such as doxycycline, chlorhexidine (CHX), and tetracycline within HNTs, leading to a more sustained release compared to free forms or those incorporated in alternative delivery systems [19–23].

CHX, a broad-spectrum antimicrobial agent with low cytotoxicity, offers promise for incorporation into acrylic resins. However, the amount of CHX included is critical as excessive levels can compromise the resin's mechanical properties and potentially exhibit cytotoxicity [24, 25]. An ideal scenario would involve a controlled release system for CHX within the resin, enabling localized action at the application site. This approach could minimize the total CHX needed, mitigating cytotoxicity risks while maintaining antimicrobial efficacy and preserving the resin's integrity. This is particularly relevant for applications such as PSIO appliances, where acrylic surfaces can harbor microbes and pose a challenge for immunocompromised patients susceptible to infections. The development of controlled release systems for antimicrobials within the oral cavity holds significant value for improving patient outcomes. To address this challenge, this study pioneers the development of PMMA-based PSIO appliances with integrated antimicrobial properties.

Therefore, the aim of this study was to synthesize a CHX-HNT-modified acrylic resin with inherent antimicrobial properties and subsequently evaluate its effectiveness in inhibiting microbial biofilm formation using a crystal violet (CV) assay while also assessing its mechanical properties.

## 2. Materials and Methods

### 2.1. Materials and Reagents

Self-polymerizing PMMA resin (Orthocryl, Dentraum) was used to fabricate acrylic specimens for this study. The specimens were further modified by incorporating chlorhexidine digluconate solution and halloysite nanotubes (Sigma-Aldrich, Germany) to create the nanocomposite material.

### 2.2. Overview

The complete timeline of this experimental *in vitro* study was 12 weeks. Acrylic resin samples were divided into five groups: one control group with no modifications, and four groups containing increasing concentrations of chlorhexidine (1%, 1.5%, 3.5%, and 4.5 wt.%). The researchers assessed the efficacy of these modified resins in inhibiting biofilm formation by *Candida albicans* (*C. albicans*), *Staphylococcus aureus* (*S. aureus*), *Streptococcus pneumoniae* (*S. pneumoniae*), and *Streptococcus agalactiae* (*S. agalactiae*) using a microtiter plate method. In addition, the mechanical properties of the resins, including flexural strength, surface roughness, and hardness, were evaluated.

### 2.3. Synthesis and Characterization of Chlorhexidine-Halloysite Nanotubes

For the synthesis of chlorhexidine-halloysite nanotubes, 20% chlorhexidine digluconate solution (CHX) was diluted to 10% by dissolving it in double-distilled water (2.5 ml CHX/2.5 ml H_2_O). This solution was then stored in darkness at room temperature. Following a published method [19, 26], 1.25 g of HNTs were mixed with 5 ml of 10% CHX solution. The mixture was vortexed for 20 seconds and then sonicated for 2 hours to ensure the CHX was evenly distributed throughout the HNTs (encapsulation). The CHX-HNTs solution was then dried in a vacuum oven for an hour. Finally, the mixture was centrifuged twice at 3500 rpm at ambient temperature, with vortexing in between. The resulting CHX-HNT nanocomposite was dried in an incubator and stored for further testing.

### 2.4. Characterization Methods

Field emission scanning electron microscopy (FE-SEM) (Inspect F50, FEI, Holland) operated at an accelerating voltage of 2 kV was used to observe the morphological topography of the HNTs, and to ascertain the level of CHX-HNT dispersion within the PMMA. In order to determine the elemental composition of the samples more accurately, energy-disperse x-ray spectroscopy (EDS) spectra and elemental mappings of the prepared samples were performed. Raman spectroscopy was performed on a Raman spectrometer (532 nm preconfigured Raman spectrometer system) with an excitation wavelength of 532 nm to identify molecules and evaluate functional groups with intramolecular bonds of CHX and HNT before and after loading. Finally, X-ray diffraction (XRD, DX2700BH, Haoyuan Instrument Co., Ltd., Dandong, China) with Cu K radiation at 40 kV, 30 mA, a 2 range of 20–100°, and a step size of 0.02° was used to acquire the diffraction patterns of HNTs, CHX, and CHX/HNTs samples. This analysis aided by X'Pert Highscore Plus software allowed researchers to confirm the successful loading of CHX onto the HNTs by identifying their characteristic crystalline patterns.

### 2.5. Preparation of Acrylic Resin Nanocomposite Samples

The experiment utilized a self-polymerizing acrylic resin for both control and experimental specimens. The acrylic resin was prepared according to the manufacturer's instructions, mixing the liquid and powder in a specific ratio (2.5 g of polymer to 1 ml of monomer). For the experimental group, CHX-HNT filler was added at various percentages into the acrylic powder before mixing it with the liquid monomer. The mixture was then vortexed to ensure even distribution of the nanotubes. After thorough mixing to a doughy consistency, the resin was shaped in molds and cured using hydroflask at 2.2 bar with 40°C water for 15 minutes to remove porosity and accelerate curing. Any excess material was removed with a standard tungsten carbide bur, and both surfaces of the samples were polished with sandpaper. Finally, the dimensions of each specimen were precisely measured with a micrometer, and all samples were sterilized using ultraviolet light for 30 minutes.

### 2.6. Microbiology

#### 2.6.1. Bacterial Strains

The researchers investigated the effectiveness of CHX-HNT nanocomposites in inhibiting biofilm formation by four types of microorganisms: *C. albicans*, *S. aureus*, *S. pneumoniae*, and *S. agalactiae*. All the investigated microbes were isolated from clinical samples, such as mucosal swabs, body fluids, and pus, from patients at the Department of Microbiology, Saad Al-Witry Specialized Hospital, Iraq. The study received ethical approval from the Institutional Ethics Committee. *C. albicans* was cultured in Sabouraud dextrose agar, incubated aerobically at 37°C for 24 hours, and then kept at 4° for later investigations. The *S. aureus* isolates were inoculated into blood agar, which was prepared according to the manufacturer's recommendations, and incubated in an aerobic condition for 48 hours at 37°. *S. pneumonia* and *S. agalactiae* isolates were inoculated on Mueller–Hinton agar supplemented by 5% sheep blood anaerobically under CO_2_ for 24 hours using a candle jar. Confirmatory tests were carried out to identify microorganisms by cultural characteristics using both standard biochemical reactions and Vitek tests.

#### 2.6.2. Microtiter Plate Method (MTP) Biofilm Production

**Figure 1:**
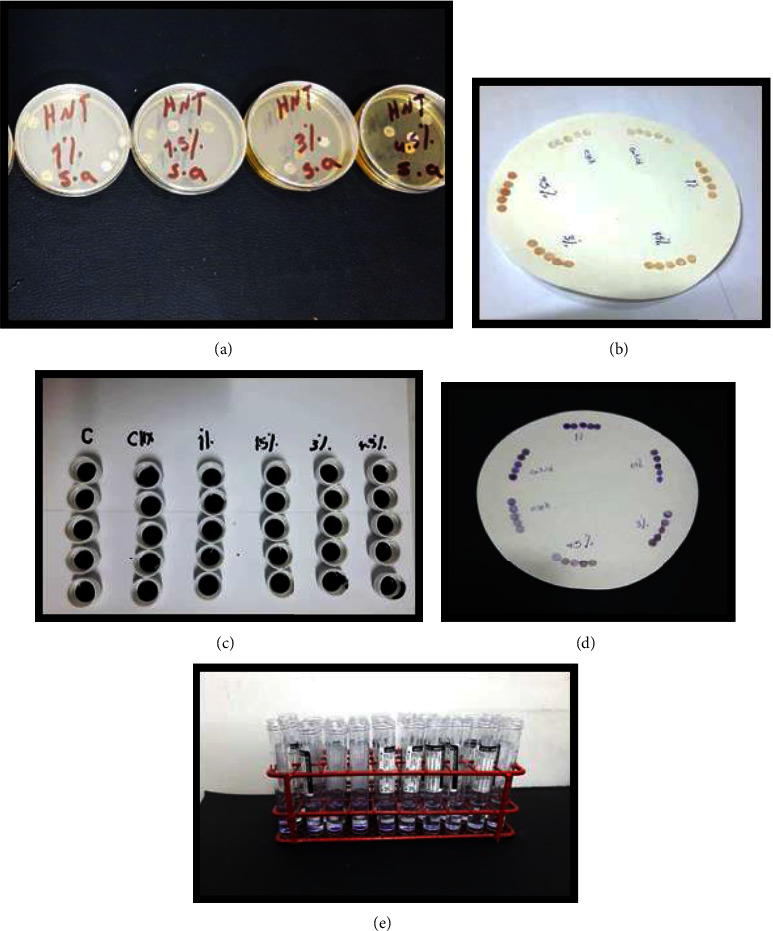
Microtiter plate test: (a) washing planktonic cells after 24 hrs of incubation, (b) drying samples, (c) crystal violet pigmentation, (d) samples after pigmentation, and (e) washing samples in ethanol.

Biofilm inhibition on the tested microbes was evaluated using a CV assay on a sterile 96-well microtiter plate, as described previously [27]. In brief, three to five microbial colonies were suspended in 4 ml of 0.85% (w/v) NaCl solution and compared with a 0.5 McFarland scale (equivalent to 1.5 × 10^8^ CFU·ml^−1^). A colony of each isolate was inoculated into tubes containing 2 ml of brain heart infusion broth (BHIB) and incubated at 37°C for 24 hours. Ten acrylic discs from each test group, measuring 0.5 mm thick and 6 mm in diameter, were immersed in wells of a 96-well microtiter plates containing a microbial suspension. Then microtiter plates were sealed and incubated at 37°C to create the microbial biofilms. After 24 h, the planktonic microorganisms with weak attachments were removed by rinsing for 1 minute with sterile saline (PBS; pH 7.4). Each specimen was stained with 0.1% (w/v) crystal violet for 20 minutes at room temperature before being rinsed twice with distilled water. For 20 minutes, the adhering biofilm was dissolved in 200 *μ*L of 95% (v/v) ethanol, as seen in Figure 1. The dye associated with the biofilm was solubilized in 33% (v/v) acetic acid, and the optical density of each group was measured with a spectrophotometer (Apel PD-303, Japan). The bacterial inhibition percentage was calculated according to the following equation [28]:

(1) $$\begin{array}{c} \text{Percentage inhibition}=100\;-\;\left[\left\{\frac{\text{OD}620\text{ of cells treated with CHX}-\text{HNTs }}{\text{OD}620\text{ of non}-\text{treated control cells}}\right\}\times 100\right]. \end{array}$$

### 2.7. Measurement of Physical and Mechanical Properties

#### 2.7.1. Flexural Strength

The flexural strength test was done according to ASTM D790-86. Rectangular acrylic bars measured (65 mm × 10 mm × 2.5 mm) in length, width, and thickness, respectively, were prepared for both the control and experimental acrylic specimens. All specimens were stored in deionized water at 37°C for 48 hours prior to investigation in order to reduce the free residual monomer, and then removed and air dried. A Tinius Olsen 330-012 3-point flex testing machine with a displacement rate of 1.0 mm/min, and a span of 40 mm was used to perform the test. Five samples from each group were used to evaluate flexural strength using the following equation and expressed in MPa:

(2) $$\begin{array}{c} \text{Flexural strength}=\frac{3PI}{2bd2}, \end{array}$$

where *P* is the load at fracture (N), *I* is the span length, *b* is the specimen's width (mm), and *d* is the specimen's height (thickness mm), while P/Y is the slope of the linear part of the stress-strain curve within the elastic portion.

#### 2.7.2. Surface Roughness

The surface topography was verified by using atomic force microscopy (AFM workshop/TT-2, USA) in tapping mode to reduce potentially damaging forces caused by contacts between the tip and the specimen. All samples were scanned with a 10 *μ*m × 10 *μ*m field of view to ensure even surface coverage. First, acrylic specimens were stored in deionized water at 37°C for 48 hours before being tested. The specimens were placed on the device's stable stage, and the location of the tested area was selected. The analyzer then traversed along each tested area at six different points. Topographical analysis was performed by importing the resulting AFM data files into the software to calculate the average roughness (Ra) and compare it among specimen groups. All of the roughness data presented in this study is an average of five scans.

#### 2.7.3. Surface Hardness

Following a standardized protocol [29], the surface hardness of the specimens was measured. First, the samples submerged in deionized water at 37°C for 48 hours. Afterward, a durometer hardness tester (TR 220, China, Shore D hardness) was used to determine the surface hardness. This involved pressing an indenter firmly and swiftly onto the sample surface and recording the highest reading. This measurement was repeated five times on different areas of each specimen, and the final value reported was the average of these five readings.

### 2.8. Statistics

Statistical analysis was performed using SPSS version 25. Descriptive statistics, including the mean, standard deviation, and minimum and maximum strength values, were calculated for each data of the experimental groups. A one-way ANOVA was done to evaluate if there were significant differences in the means of the various experimental groups. The Tukey test was employed at the chosen level of probability (*p* < 0.05) to determine if the means were significantly different from each other.

## 3. Results

### 3.1. Structural and Morphological Characterization

#### 3.1.1. Field Emission Scanning Electron Microscope

**Figure 2:**
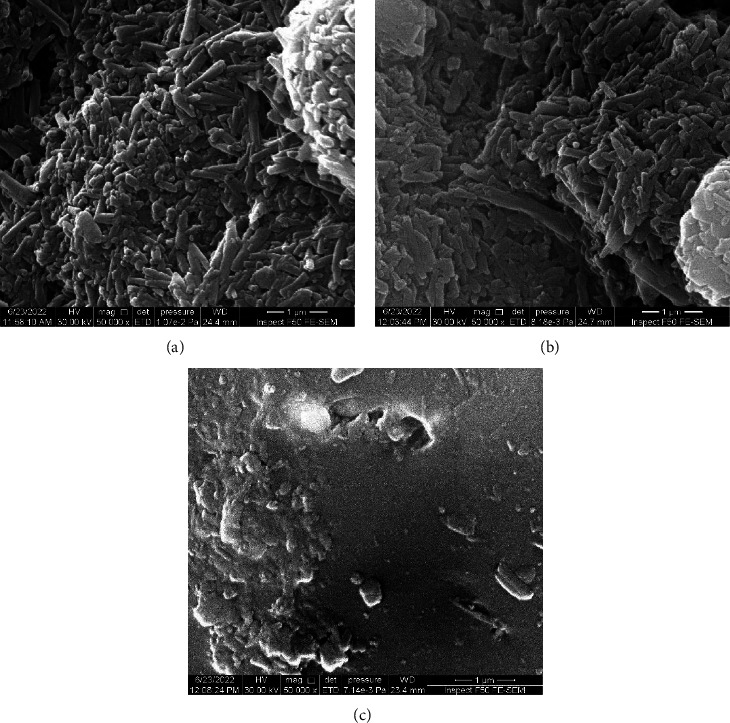
FE-SEM images: (a) halloysite nanotubes, (b) halloysite loaded with CHX, and (c) acrylic surface incorporated with HNTs-CHX.

FE-SEM images at different magnification powers of HNTs before CHX loading shown in Figure 2 confirmed the tubular geometry of HNTs with a relative heterogeneous particle size distribution having a mean diameter of approximately 70 nm and 400 nm in length. While FE-SEM images of CHX-HNT nanocomposites after acrylic incorporation confirmed the fair dispersion with few aggregations of the nanofiller within the PMMA acrylic matrix.

#### 3.1.2. Characterization by EDX Spectroscopy

**Figure 3:**
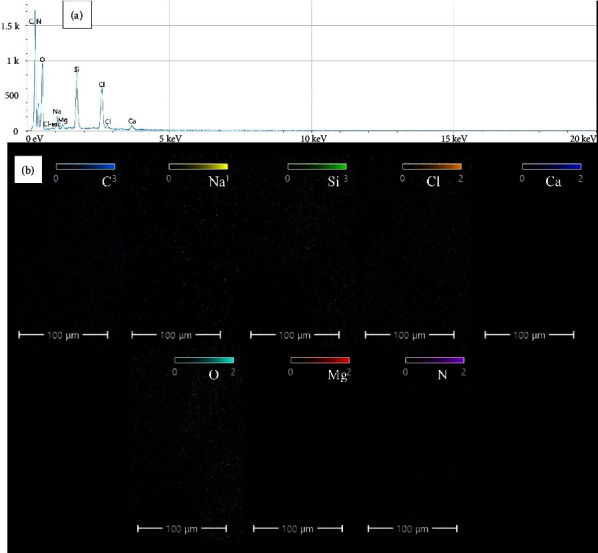
(a) EDS and (b) elemental mapping results for the CHX sample.

**Figure 4:**
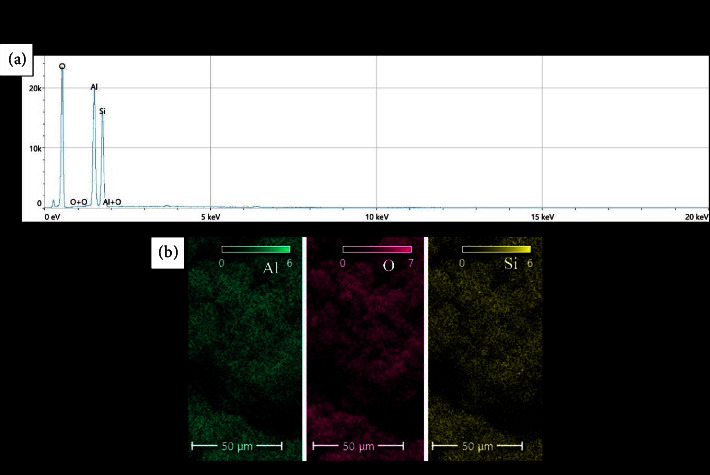
(a) EDS and (b) elemental mapping results for the HNT sample.

**Figure 5:**
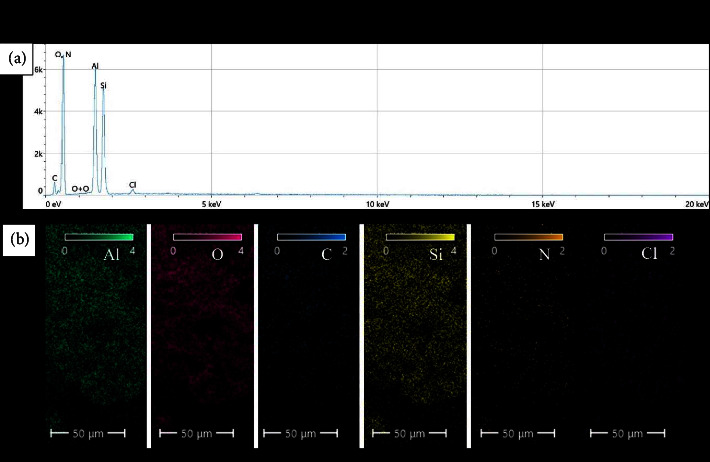
(a) EDS and (b) elemental mapping results for the CHX-HNT sample.

In order to determine the elemental composition of the samples accurately, the EDS spectra and elemental mappings of the prepared samples were performed. The results of EDS and elemental mapping analyses for the CHX, HNT, and CHX-HNT samples are shown in Figures 3–5, respectively.

The molecular formula of pure chlorhexidine gluconate is C_34_H_54_Cl_l2_N_10_O_14_ [30]. From Figure 3, it can be seen that carbon, oxygen, nitrogen, and chlorine are the main elements detected for the CHX sample, which are the main elements in the chlorhexidine gluconate molecular structure. It should be noted that hydrogen cannot be detected with the EDS analysis because it has a very low atomic number and does not emit X-rays with sufficient energy to be detected by the EDS detector. The EDS detector is designed to detect X-rays emitted by elements with higher atomic numbers, typically ranging from boron to uranium. In addition, hydrogen has a very low electron density, making it difficult to generate X-rays through interactions with the electron beam used in the EDS analysis. Furthermore, silicon, sodium, calcium, and magnesium elements were also detected (with a total atomic percentage less than 4%) as impurities. Elemental mapping (Figure 3(b)) shows that the elements are well distributed throughout the surface.

The main elements found in the HNT sample were Si, O, and Al, indicating the presence of halloysite nanotubes with a chemical structure of Al_2_Si_2_O_5_(OH)_4_. The theoretical atomic percentages for oxygen, silicon, and aluminum atoms (ignoring the hydrogen atom) are about 70%, 15%, and 15%, which are close to those derived from experimental data (about 72%, 14%, and 14% for O, Si, and Al, respectively) as shown in Figure 4(a).

The analysis of the CHX-HNT nanocomposite confirms its successful synthesis. This is because the presence of elements from both original materials (Al, O, and Si from HNTs and C, N, and Cl from CHX) is detected. Furthermore, impurities such as magnesium, sodium, and calcium found in the initial CHX sample are absent in the final nanocomposite, indicating their removal during synthesis. Interestingly, the data reported in Figure 5(a) suggest the nanotubes (HNTs) are the dominant phase (10 times more than CHX), acting as a scaffold for the CHX molecules. This is further supported by the mapping results (Figure 5(b)), where some areas lack carbon, chlorine, and nitrogen, implying these regions are the HNT nanotubes decorated by the CHX molecules on the outer surface.

#### 3.1.3. Raman Spectroscopy

**Figure 6:**
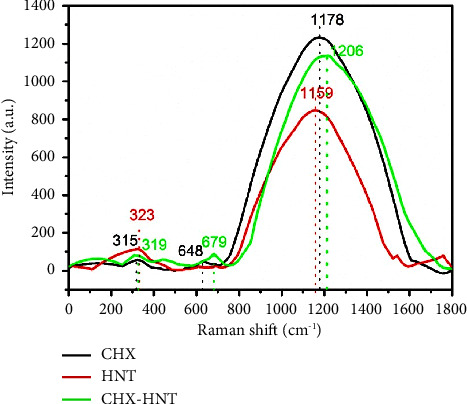
Raman spectra of CHX, HNT, and CHX-HNT samples in the Raman shift range of 0–1800 cm^−1^.

Raman spectroscopy is used in chemistry to identify molecules and study chemical bonding and intramolecular bonds [31, 32].  Figure 6 shows the Raman spectra of CHX, HNT, and CHX-HNT samples in the Raman shift range of 0–1800 cm^−1^.

In the Raman spectrum of CHX, a broad band can be seen in the Raman shift range of 800–1600 cm^−1^ with a maximum point at 1178 cm^−1^. This broad peak can be assigned to overlapped peaks of stretching vibration, of asymmetric stretching vibration of O-CC and asymmetric rocking vibration of C-H bonds. In addition, two weak peaks can be seen at 323 cm^−1^ and 642 cm^−1^ due to bending vibration of CCN and deformation of N-H bonds (amid group) in the chemical structure of chlorhexidine gluconate, respectively [33, 34]. The Raman spectrum of HNT shows three peaks at 316 cm^−1^ and 1159 cm^−1^ corresponding to Al-OH bending vibration and Si-O stretching vibration, respectively [35, 36]. From the Raman spectrum of the CHX-HNT nanocomposite, it is clear that the Raman peaks for both structures in their spectra appeared again in the Raman spectrum of the CHX-HNT nanocomposite, confirming the successful synthesis of the nanocomposite. However, it can be seen that the peak position of the deformation of N-H and stretching vibration of O-CC/Si-O bonds shifted to more Raman shifts (679 cm^−1^ and 1206 cm^−1^, respectively) due to the interactions between the chemical structures of the nanocomposite's components.

#### 3.1.4. X-Ray Diffraction Analysis

**Figure 7:**
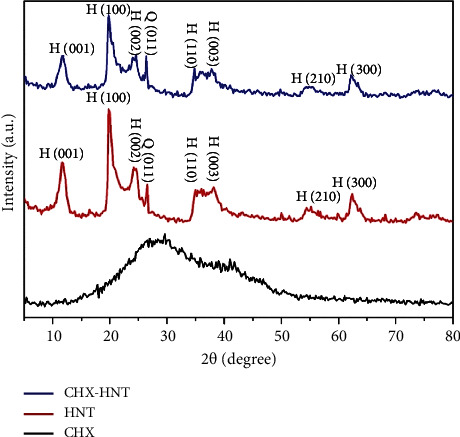
XRD patterns of CHX, HNT, and CHX-HNT samples in the 2*θ* range of 5°–80°.

X-ray diffraction (XRD) analysis was employed to analyze the crystalline structures of CHX, HNTs, and the resulting CHX-HNT nanocomposite. XRD is a well-established technique for determining the arrangement of atoms within a crystalline material and can reveal any structural modifications that occur during the formation of the nanocomposite (Figure 7).

It can be observed that the XRD pattern of the CHX sample has a predominantly amorphous profile with a broad peak in the 2*θ* range of 10°–50°, as was seen previously for this structure [37, 38]. For the XRD pattern of the HNT sample, by coinciding the peaks with the reference diffraction patterns using the diffractor software, it was found that the meta-halloysite phase with the chemical formula of Al_2_Si_2_O_5_(OH)_4_ and the JCPDS No. 00-029-1487 reference code was the main crystalline structure in the sample. According to the reference card, the crystalline phase has a hexagonal crystal structure with a space group of P. In this pattern, the diffraction planes are (001), (100), (002), (110), (003), (210), and (300) appeared at 2*θ* = 11.6°, 19.9°, 24.2°, 35.9°, 38.2°, 55.2°, and 62.4°, respectively. The peak at 2*θ* = 26.5° can be attributed to the (011) plane of quartz crystalline phase, which usually present as an impurity in halloysite nanotubes [39]. The crystallite size of the sample was calculated using the Scherrer equation [40]:

(3) $$\begin{array}{c} D=\frac{K\;\lambda }{\beta }\cos\;\theta , \end{array}$$

where *D* is the mean dimension of the homogeneous crystallites along an axis perpendicular to the hkl system considered, *β* is the width of the diffraction profile (the full width at half maximum, FWHM), *K* is the Scherrer constant approximation close to unity, and *λ* is the corresponding X-ray wavelength (units are 2*θ* scale in radians). According to the equation, the crystallite size for the HNT sample based on the sharpest peak (at 2*θ* = 19.9°) was about 11 nm.

The XRD analysis of the CHX-HNT nanocomposite indicated the preservation of the HNT's crystalline structure, evidenced by the presence of diffraction peaks at similar positions to pristine HNTs. However, these peaks exhibited broadening, suggesting a decrease in crystallinity likely due to the incorporation of the amorphous CHX phase, as previously observed by Wu et al. [41].

### 3.2. Biofilm Inhibition Test

Microbiological analysis revealed biofilm formation by all isolates tested.  Table 1 presents the results of a normality test and descriptive statistics for the biofilm inhibition efficacy of CHX-HNTs. The Shapiro–Wilk test confirmed normal distribution of all data. Notably, CHX-HNTs demonstrated significant antibiofilm activity against all investigated strains, including *C. albicans*, *S. aureus*, *S. pneumoniae*, and *S. agalactiae*.

**Figure 8:**
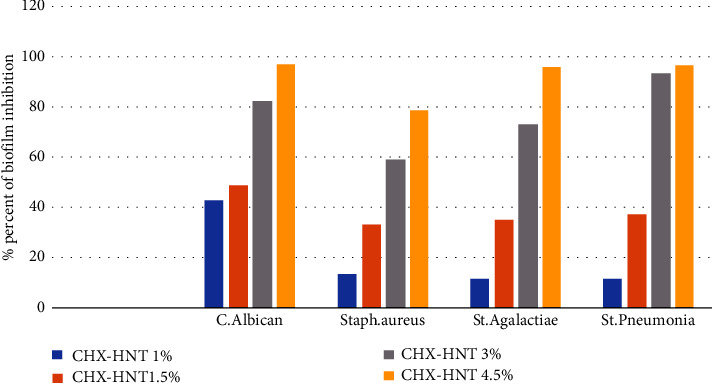
Effect of different percentages of CHX-HNT incorporation of acrylic samples on microbial biofilm formation.

The statistical analysis of data presented in Tables 2 and 3 revealed a dose-dependent inhibitory effect of CHX-HNTs on biofilm formation by all investigated pathogens. The application of CHX-HNTs at concentrations ranging from 1 wt.% to 4.5 wt.% resulted in a statistically significant decrease in biofilm formation (*p* < 0.05), as seen in Figure 8.

### 3.3. Mechanical Properties

Table 4 summarizes the mechanical properties of the control and experimental groups. The statistical analysis revealed no significant increase (*p* > 0.05) in flexural strength upon incorporation of CHX-HNTs into the acrylic resin. Interestingly, the unmodified PMMA control exhibited the highest flexural strength among all groups, although this difference was not statistically significant. These results suggest a potential negative impact of CHX-HNT filler on flexural strength, with the group containing the highest concentration (4.5 wt.%) displaying the weakest flexural strength compared to others.

**Figure 9:**
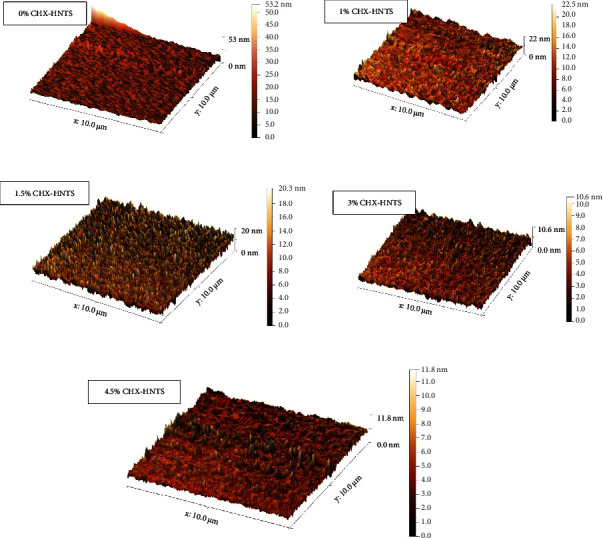
AFM images of the surface topography of PMMA denture base specimens with different HX-HNT fillers loading (scan of 10 × 10 *μ*m).

Consistent with the flexural strength results, CHX-HNT incorporation resulted in a slight, yet statistically insignificant (*p* > 0.05) increase in surface roughness of the acrylic resin (Figure 9). This trend suggests a potential correlation between filler concentration and surface roughness. However, the hardness measurements revealed no significant difference between the control and experimental groups, indicating minimal impact of CHX-HNTs on this mechanical property.

## 4. Discussion

CLP patients' oral flora has an increased prevalence of potentially pathogenic microbial colonization, including *Candida* species, *S. aureus*, *S. pneumonia*, and *S. agalactiae* that can lead to serious infections and pose challenges during postsurgical healing due to the immunocompromised state of CLP patients [42]. To mitigate these risks, meticulous oral hygiene practices and regular preventive dental care are crucial components of CLP treatment protocols [41, 43]. However, current methods for cleaning removable acrylic dentures, often relying solely on mechanical and chemical means, have proven inadequate in eliminating these contaminating microorganisms [44–46]. Therefore, there is a growing demand for an affordable, easy-to-implement strategy for preventing the accumulation of dental plaque on these prosthetic devices [47–49]. In an attempt to combat microbial adhesion on acrylic resin, this study implemented a novel approach by incorporating CHX-HNT nanocomposite fillers. This study used a novel method to modify acrylic base materials by adding CHX-HNT fillers in an attempt to potentially discourage microbial adhesion. The first objective of this research was to develop and characterize a CHX-HNT nanocomposite, and second, to evaluate the impact of integrating this nanocomposite at various concentrations on both its ability to inhibit biofilm formation and the mechanical properties of the acrylic resin.

As an alternative to more expensive nanofillers such as carbon nanotubes (CNTs), HNTs have gained growing research interest due to their lower cost in the manufacturing of high-performance materials such as polymers [50]. HNTs are characterized by a hydroxyl with a lower level of density that permits smooth diffusion in a polymer matrix compared to other nanoclays. The unique high aspect ratio tubular structure and the polarity of the tubule surface show that the HNTs are suitable candidates to secure generous dispersal in the polymer matrices [18, 23, 51]. Due to the critical role of appropriate nanotube morphology in both functionality and successful nanocomposite synthesis [52], a comprehensive characterization of the HNTs was undertaken. This involved employing various structural analysis techniques such as FE-SEM, EDS, Raman spectroscopy, and XRD. Our analysis confirmed that the morphology and size of the HNTs matched those reported in previous studies [21, 53, 54]. In addition, these techniques successfully verified the homogenous distribution of CHX-HNTs within the acrylic matrix, with minimal instances of localized nanofiller aggregation.

CHX was selected among other antimicrobial reagents as it has established itself as the leading agent for chemical plaque control. CHX exhibits ability to inhibit adherence of microorganisms to a surface thereby preventing growth and development of biofilms [55, 56]. Numerous studies have documented its efficacy in inhibiting microbial growth, dental plaque and gingivitis, particularly when used as a mouthwash [55–57]. Notably, Redding et al. demonstrated that CHX exhibited the highest rate of inhibition against *C. albicans* biofilm formation on denture materials compared to other antifungal drugs like Amphotericin B and nystatin [55]. Our investigation confirms the effectiveness of CHX-HNTs as an antiplaque agent. We observed biofilm inhibition rates exceeding 98% against *C. albicans*, *S. pneumoniae*, and *S. agalactiae*. Furthermore, a statistically significant decrease in biofilm formation was observed with increasing concentrations of the CHX-HNTs nanofiller. These findings align with those reported by Al-Mousawi and Alhuwaiz who evaluated the biofilm inhibitory efficacy of CHX-hexametaphosphate against *C. albicans* [58]. Their study revealed that at 5 mM concentration, the antibiofilm activity could reach nearly 100% inhibition, with a gradual decrease in efficacy observed at lower nanoparticle concentrations [58]. Reinforcing the rationale for CHX selection, Alvendal et al. also highlighted its superior efficacy against *C. albicans* biofilms compared to fluconazole, particularly in eradicating pre-existing biofilms [59]. This is crucial as *Candida* spp. are increasingly implicated in biomaterial-associated infections, both orally and systemically. These fungal pathogens readily adhere to and colonize oral surfaces such as mucosa and denture acrylics, further complicating matters by coaggregating with other oral bacteria [60]. While CHX-HNTs effectively inhibited biofilm formation of various investigated pathogens, *S. aureus* presented a different challenge. Although CHX-HNTs significantly reduced *S. aureus* colonization (86%), complete eradication was not achieved as supported by previous studies [61, 62]. This suggests that *S. aureus* biofilms may require a longer exposure time to CHX for complete eradication. Supporting this notion, research by Toté et al. in Belgium demonstrated that extended contact time with chlorhexidine enhances its antibiofilm activity against *S. aureus* [63].

Considering the mechanical properties of the nanofiller-modified acrylic, research findings revealed that while the incorporation of CHX-HNTs in acrylic resin provided satisfactory inhibition of microbial adhesion, it had no significant effect on the materials' properties (flexural strength, roughness, and hardness). A slight, dose-dependent decrease in flexural strength and a corresponding increase in surface roughness were observed. This can be attributed to the inherent hydrophilicity and high surface area of halloysite nanotubes, which may promote filler agglomeration at higher concentrations [22, 23]. These agglomerates can act as stress concentrators within the composite, compromising strength and limiting elastic modulus enhancement in dental resins [64, 65]. The observed increase in surface roughness is likely due to the presence of nanoparticles on the acrylic specimen surface [66]. Similar results were reported by Feitosa et al. who explored CHX-HNT applications in dental adhesives [67]. Their study demonstrated that CHX-loaded nanocomposite adhesives exhibited sufficient antibacterial activity while maintaining essential mechanical properties, including the degree of conversion, microhardness, water sorption, and biocompatibility. However, it is important to note that Barot et al. observed contrasting results [22]. Their study found that incorporating CHX-HNTs into dental resin composites actually enhanced both mechanical properties and antibacterial activity. This difference highlights the potential influence of the specific material being integrated with CHX-HNTs.

The incorporation of HNTs into PMMA resin can improve hardness by causing the HNT filler that is not strongly adhered to the resin to disperse or dissociate under load, creating frictional force that allows stress distribution across the matrix cracks and thereby increasing the material's resistance to indentation [66–69]. The FTIR study results confirmed the absence of a chemical interaction between the acrylic resin and the CHX/HNTs filler, validating the hardness results found because the presence of the CHX/HNTs caused no statistically significant change in the acrylic resin property.

### 4.1. Limitations

This is an *in vitro* experimental study. Thus, the complexity of the biofilm community, the lack of knowledge regarding the identity and abundance of each biofilm resident, and the lack of salivary and host factors hinder our ability to precisely replicate the intraoral scenario. The authors acknowledge that not all processes during biofilm formation can be adequately simulated with a laboratory approach; thus, the results of this study should lead to further *in situ* and *in vivo* studies to validate their use.

### 4.2. Strength of the Study

This study set out to strengthen the early oral health preventative and maintenance programs in CLP patients. Our research will add to the existing body of knowledge by being the first to construct a CLP infant orthopedic appliance with an antimicrobial property by successfully incorporating chlorhexidine-halloysite nanotubes into a PMMA resin. Results of this study showed that incorporation of CHX-HNT into PMMA-based resins allowed for its subsequent release from the matrices, which enabled the resins to inhibit *C. albicans* and bacterial biofilm without affecting the appliance's mechanical properties, demonstrating promising antimicrobial activity.

## 5. Conclusions

The CHX-HNT-modified acrylic resin exhibited remarkable efficacy in inhibiting biofilm formation while maintaining satisfactory mechanical properties. These findings suggest its promise as a novel preventive strategy for managing infectious complications in cleft lip and palate infants, potentially improving disease control in this patient population.

## Figures and Tables

**Table 1:** Descriptive statistics of the biofilm inhibition ability of the CHX-HNTs.

Microbes	Material (%)	Descriptive statistics			Shapiro–Wilk	
		Min	Max	Mean	S.D	*p* value
*C. albicans*	CHX-HNT 1	37.00	53.00	42.8000	6.26099	0.272
	CHX-HNT 1.5	39.00	59.00	49.0000	7.31437	0.985
	CHX-HNT 3	78.00	87.00	81.8000	3.42053	0.617
	CHX-HNT 4.5	95.00	99.00	96.6000	1.67332	0.269

*S. aureus*	CHX-HNT 1	6.00	19.00	13.6000	5.41295	0.314
	CHX-HNT 1.5	27.00	44.00	33.2000	7.52994	0.335
	CHX-HNT 3	51.00	66.00	59.0000	5.61249	0.833
	CHX-HNT 4.5	74.00	86.00	79.0000	4.89898	0.314

*S. agalactiae*	CHX-HNT 1	7.00	18.00	11.8000	5.06952	0.325
	CHX-HNT 1.5	25.00	43.00	35.2000	7.46324	0.619
	CHX-HNT 3	63.00	81.00	73.0000	7.24569	0.246
	CHX-HNT 4.5	94.00	98.00	95.8000	1.48324	0.786

*S. pneumonia*	CHX-HNT 1	10.00	21.00	11.8000	8.46759	0.314
	CHX-HNT 1.5	27.00	44.00	37.4000	6.42651	0.335
	CHX-HNT 3	92.00	94.00	93.2000	1.09545	0.833
	CHX-HNT 4.5	95.00	99.00	96.6000	1.51658	0.314

^*∗∗*^Statistically significant difference (*p* value ≤0.005); min: minimum; max: maximum; SD: standard deviation.

**Table 2:** ANOVA test of the biofilm inhibition test.

Microbes	ANOVA	Sum of squares	d.f	Mean square	*F*-test	Sig
*C. albicans*	Between groups	28270.160	4	7067.540	329.643	0.000
	Within groups	428.800	20	21.440		
	Total	28698.960	24			

*S. aureus*	Between groups	20894.960	4	5223.740	184.584	0.000
	Within groups	566.000	20	28.300		
	Total	21460.960	24			

*S. pneumonia*	Between groups	40658.000	4	10164.500	436.245	0.000
	Within groups	466.000	20	23.300		
	Total	41124.000	24			

*S. agalactiae*	Between groups	32854.960	4	8213.740	301.754	0.000
	Within groups	544.400	20	27.220		
	Total	33399.360	24			

**Table 3:** Inferential statistics of the biofilm inhibition test using Tukey's test.

*C. albicans*	CHX-HNT CONC	Sig	*S. aureus*	CHX-HNT CONC	Sig	*S. agalactiae*	CHX-HNT CONC	Sig	*S. pneumonia*	CHX-HNT CONC	Sig
Acrylic	CHX	0.000	Acrylic	CHX	0.000	Acrylic	CHX	0.000	Acrylic	CHX	0.000
	1.00%	0.000		1.00%	0.001		1.00%	0.013		1.00%	0.000
	1.50%	0.000		1.50%	0.000		1.50%	0.000		1.50%	0.000
	3.00%	0.000		3.00%	0.000		3.00%	0.000		3.00%	0.000
	4.50%	0.000		4.50%	0.000		4.50%	0.000		4.50%	0.000

CHX	1.00%	0.000	CHX	1.00%	0.000	CHX	1.00%	0.000	CHX	1.00%	0.000
	1.50%	0.000		1.50%	0.000		1.50%	0.000		1.50%	0.000
	3.00%	0.805		3.00%	0.000		3.00%	0.000		3.00%	0.530
	4.50%	0.045		4.50%	0.004		4.50%	1.000		4.50%	1.000

CHX-HNT 1%	1.50%	0.435	CHX-HNT 1%	1.50%	0.000	CHX-HNT 1%	1.50%	0.000	CHX-HNT 1%	1.50%	0.000
	3.00%	0.000		3.00%	0.000		3.00%	0.000		3.00%	0.000
	4.50%	0.000		4.50%	0.000		4.50%	0.000		4.50%	0.000

CHX-HNT 1.5%	3.00%	0.000	CHX-HNT 1.5%	3.00%	0.000	CHX-HNT 1.5%	3.00%	0.000	CHX-HNT 1.5%	3.00%	0.000
	4.50%	0.000		4.50%	0.000		4.50%	0.000		4.50%	0.000

CHX-HNT 3%	4.50%	0.002	CHX-HNT 3%	4.50%	0.000	CHX-HNT 3%	4.50%	0.000	CHX-HNT 3%	4.50%	0.671

**Table 4:** Descriptive and inferential statistics of mechanical properties of different CHX-HNT acrylic samples.

Tests	Groups	Descriptive statistics				ANOVA	
		Mean	SD	Min	Max	*F*-test	*p* value
Flexural strength (MPa)	Acrylic	79	6.63	71.800	87.5	1.821	0.164
	CHX-HNT 1%	77.09	4.02	71.250	81.2		
	CHX-HNT 1.5%	76.3	10.60	65.000	87.5		
	CHX-HNT 3%	70	7.86	62.500	81.3		
	CHX-HNT 4.5%	69.3	5.57	63.700	77.5		

Roughness (Ra)	Acrylic	1.488	0.21005	1.180	1.7	0.352	0.839
	CHX-HNT 1%	1.516	0.53580	0.780	2.1		
	CHX-HNT 1.5%	1.68	0.20657	1.450	1.9		
	CHX-HNT 3%	1.67	0.63263	0.720	2.4		
	CHX-HNT 4.5%	1.810	0.69876	0.630	2.4		

Hardness	Acrylic	21.43	0.93095	20.000	23	1.047	0.408
	CHX-HNT 1%	21.2	0.87407	20.000	22		
	CHX-HNT 1.5%	22.2	1.1506	21.000	24		
	CHX-HNT 3%	21.1	0.93897	20.000	22		
	CHX-HNT 4.5%	21.4	0.1.01735	20.000	23		

^*∗∗*^Statistically significant difference (*p* value ≤0.005); min: minimum; max: maximum; SD: standard deviation.

## Data Availability

The data used to support the findings of this study are included within the article.
